# Improvements in fetal heart rate analysis by the removal of maternal-fetal heart rate ambiguities

**DOI:** 10.1186/s12884-015-0739-1

**Published:** 2015-11-19

**Authors:** Paula Pinto, Cristina Costa-Santos, Hernâni Gonçalves, Diogo Ayres-De-Campos, João Bernardes

**Affiliations:** Hospital Nélio Mendonça, Funchal, Portugal; Departamento de Obstetrícia e Ginecologia, Faculdade de Medicina da Universidade do Porto, Porto, Portugal; Center for Health Technology and Services Research (CINTESIS), Porto, Portugal; Centro Hospitalar São João, Porto, Portugal; INEB – Institute of Biomedical Engineering; I3S – Institute for Research and Innovation in Health, University of Porto, Porto, Portugal; Unidade Local de Saúde de Matosinhos, Hospital Pedro Hispano, Matosinhos, Portugal

**Keywords:** Heart rate, Fetal, Maternal heart rate, Cardiotocography, Misinterpretation, Computer analysis, FIGO guidelines

## Abstract

**Background:**

Misinterpretation of the maternal heart rate (MHR) as fetal may lead to significant errors in fetal heart rate (FHR) interpretation. In this study we hypothesized that the removal of these MHR-FHR ambiguities would improve FHR analysis during the final hour of labor.

**Methods:**

Sixty-one MHR and FHR recordings were simultaneously acquired in the final hour of labor. Removal of MHR-FHR ambiguities was performed by subtracting MHR signals from their FHR counterparts when the absolute difference between the two was less or equal to 5 beats per minute. Major MHR-FHR ambiguities were defined when they exceeded 1 % of the tracing. Maternal, fetal and neonatal characteristics were evaluated in cases where major MHR-FHR ambiguities occurred and computer analysis of FHR recordings was compared, before and after removal of the ambiguities.

**Results:**

Seventy-two percent of tracings (44/61) exhibited episodes of major MHR-FHR ambiguities, which were not significantly associated with any maternal, fetal or neonatal characteristics, but were associated with MHR accelerations, FHR signal loss and decelerations. Removal of MHR-FHR ambiguities resulted in a significant decrease in FHR decelerations, and improvement in FHR tracing classification.

**Conclusions:**

FHR interpretation during the final hour of labor can be significantly improved by the removal of MHR-FHR ambiguities.

## Background

Fetal heart rate (FHR) monitoring may be affected by the temporary acquisition of the maternal heart rate (MHR), both when using external monitoring with Doppler ultrasound [1], and when using internal monitoring with electrocardiography (ECG) [2–6]. Inadvertent MHR acquisition with external monitoring has been reported in up to 90 % of intrapartum recordings, for an average of 6.2 % of tracing length [1], while with internal monitoring it is usually due to the detection of maternal signals transmitted via the fetal electrode in cases of fetal death [2, 5]. Overall, significant errors in FHR interpretation may occur, including missing the diagnoses of newborn acidemia [7, 8] and fetal death [2–6].

Some methods reduce the occurrence of these MHR-FHR ambiguities. FHR signal acquisition with internal [9, 10] or transabdominal [1] ECG, rather than with Doppler ultrasound, has been shown to improve signal quality, making it less prone to MHR-FHR ambiguities [1, 9, 10]. Simultaneous registration of the MHR using ECG or oximetry [7, 11] allows a comparison of MHR and FHR recordings and facilitates the detection of overlapping segments. Detection of P waves in the fetal ECG may be also helpful as a marker of fetal signals [12]. Visualization of FHR movements on ultrasound is recommended in doubtful cases, namely when fetal death is suspected [11].

While there is a reasonable amount of evidence to document cases of missed newborn acidemia and fetal death associated with MHR-FHR ambiguities, little research has been published on more subtle signal contaminations [1]. In particular, the effect of systematic cleaning of the FHR signal when MHR is suspected has, to our knowledge, not been previously evaluated.

The objective of this study was to assess the effect of removal of MHR-FHR ambiguities on the analysis of FHR recordings, during the last hour of labor. The hypothesis was that this would alter the identification of some FHR features and thus improve overall tracing classification.

## Methods

The study followed the Helsinki Declaration, was approved by the local Ethics Committee (“Comissão de Ética do SESARAM”) and all women gave their informed written consent to participate. Sixty-two consecutively acquired simultaneous recordings of MHR and external FHR, with good signal quality were selected, from the same number of labouring women, with uneventful singleton pregnancies, with fetuses in cephalic presentations, in the last recorded hour before birth (with a maximum 10-minute interval before vaginal delivery or 30 min before caesarean delivery). All but two women were under epidural analgesia on request. Labor protraction or arrest, secondary to poor uterine activity, was treated with oxytocin, according to the local protocol. No other drugs with a potential to effect MHR or FHR were administered, namely salbutamol or parasympathetic agonists.

Gestational ages were confirmed by first trimester ultrasound dating, Apgar scores were estimated by the attending healthcare professional, and umbilical artery blood pH was obtained by paired sampling immediately after birth.

For acquisition of MHR signals, an ECG sensor was connected to three electrodes positioned on the maternal thorax, and for FHR signals a Doppler ultrasound probe was placed on the maternal abdomen. One case was excluded because of poor signal quality that required conversion to internal FHR monitoring. Acquisition of signals at 1600 Hz and heart rate (HR) extraction was performed using a STAN® 31 fetal monitor (Neoventa Medical, Gothemburg, Sweden).

**Fig. 1:**
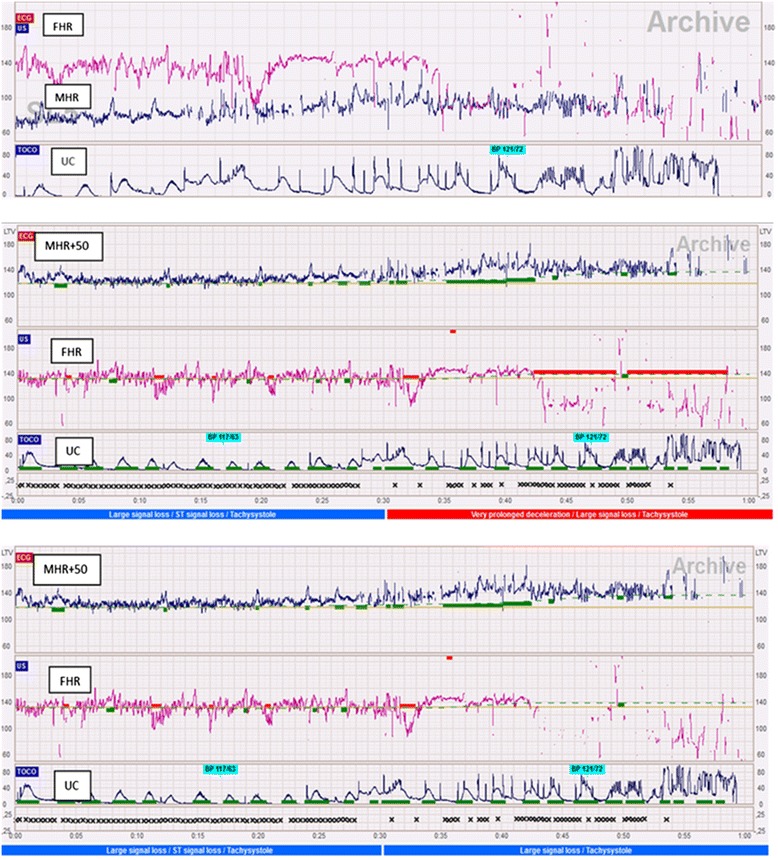
Maternal heart rate (MHR) baseline misinterpreted as a prolonged fetal heart rate (FHR) deceleration. *Top*: simultaneous recording of the last 45 min of the MHR (*black*), FHR (*pink*) and uterine contractions (UC) signals; *Middle and Bottom*: computer analysis before and after removal of MHR-FHR ambiguities. For computerized analysis of MHR tracings, a scale change (MHR + 50 beats per minute) was performed (*last hour*). After the removal of ambiguities, the FHR alarm changed from red to blue

HR, in beats per minute (bpm), was then conveyed at 4-Hz via the digital port of the fetal monitor to the Omniview-SisPorto® system (Speculum, Lisbon, Portugal) for signal recording and analysis. The system closely follows the International Federation of Gynaecology and Obstetrics (FIGO) guidelines for fetal monitoring [13–16] and has been extensively validated both in the ante [16, 17] and intrapartum periods [15, 17–19]. Analysis is carried out without signal reduction or averaging (Fig. 1). The baseline is estimated using a complex algorithm based on histogram and STV analysis, aimed at finding the HR level of stable segments with normal variability, within the physiological limits of 110–150 bpm [14–16]. STV is determined as the difference between two adjacent HR beats, and considered abnormal when lower than one beat per minute. Accelerations and decelerations are detected as HR deviations, above or below baselines, with at least 15 bpm of amplitude and 15 s duration. LTV is estimated in HR segments that do not display accelerations or decelerations, as the difference between the highest and lowest values, in a sliding window of 1 min, and is classified as abnormal when <5 bpm [13–16]. All of these basic FHR features are then used by the system to elicit an overall tracing classification, as green or blue (normal tracing), yellow (suspicious tracing) or red (pathologic tracing)) [13, 14]. The system was developed essentially for FHR analysis, but the same principles were applied in this study for analysis of the MRH, after scale conversion [13].

To remove MHR-FHR ambiguities, FHR signals were subtracted of their MHR counterparts when the absolute difference between them was equal or less than 5 bpm. This threshold was based on a report by Reinhard et al. [1] and on a pilot test performed in five cases with typical MHR-FHR ambiguities detected by visual analysis. Major MHR-FHR ambiguities were defined when more than 1 % of the tracing was affected and minor ambiguities when this value was between 0 and 1 % (Table 1).

**Table 1:** Maternal, fetal, delivery and newborn characteristics associated with minor and major MHR-FHR ambiguities

	MHR-FHR ambiguity		
	Minor (*n* = 17)	Major (*n* = 44)	*p*
Maternal characteristics			
Age (years) *mean (SD)*	26 (6)	28 (5)	0.134
BMI *mean (SD)*	27 (3)	28 (3)	0.186
Multiparity *n (%)*	5 (29)	11 (25)	0.752
Labour			
Epidural *n (%)*	17 (100)	42 (95)	1.000
Mode of delivery			0.105
Cesarean *n (%)*	4 (24)	2 (4)	
Operative vaginal *n (%)*	5 (29)	18 (41)	
Normal vaginal *n (%)*	8 (47)	24 (55)	
MHR			
Signal loss (%) *median (IQR)*	9 (1 to 11)	6 (1 to 16)	0.834
Basal MHR (bpm) *mean (SD)*	80 (12)	77 (12)	0.292
Accelerations (*n*) *mean (SD)*	12 (6)	18 (10)	**0.013**
Decelerations (*n*) *median (IQR)*	0 (0 to 0.5)	0 (0 to 0)	0.913
Abnormal LTV (%) *median (IQR)*	0 (0 to 0)	0 (0 to 0)	0.705
FHR			
Signal loss (%) *mean (SD)*	13 (7)	22 (11)	**0.002**
Baseline (bpm) *mean (SD)*	140 (10)	136 (12)	0.180
Accelerations (*n*) *median (IQR)*	9 (3 to 15)	7 (4 to10)	0.415
Decelerations (*n*) *median (IQR)*	3 (1 to 7)	8 (6 to 10)	**0.002**
Prolonged decelerations (n) *median (IQR)*	0 (0 to 0)	0 (0 to 1)	0.073
Abnormal LTV (%) *median (IQR)*	3 (1 to 9)	1 (0 to 3)	0.055
Alarm *n (%)*			**0.001**
Green-blue	14 (82)	15 (34)	
Yellow-red	3 (18)	29 (66)	
Newborn			
Gestational age (weeks) *mean (SD)*	39 (1)	39 (1)	0.672
Male *n (%)*	8 (47)	26 (59)	0.396
Birthweight (grams) *mean (SD)*	3171 (366)	3254 (318)	0.384
Apgar 1 *median (IQR)*	9 (9 to 10)	9 (9 to 10)	0.530
Apgar 5 *median (IQR)*	10 (10 to 10)	10 (10 to 10)	0.298
UAB pH *mean (SD)*	7.25 (0.08)	7.24 (0.07)	0.586

*SD* standard deviation, *IQR* inter-quartile range, *BMI* body mass index

*p* values with statistical significance are displayed in bold

Maternal, fetal and neonatal characteristics associated with major and minor MHR-FHR ambiguities were evaluated (Table 2). In tracings with and without major ambiguities, computer analysis of FHR recordings before and after the deletion of ambiguities was compared, (Table 3), and the association between FHR classification and newborn umbilical artery blood pH < or ≥7.15 was assessed.

**Table 2:** Computer analysis of FHR tracings in cases with and without major MHR-FHR ambiguities, before and after their removal

	*N*	Before	After	*p*
No major MHR-FHR ambiguity				
Signal loss (%) *mean (SD)*	17	13 (7)	14 (7)	**<0.001**
Basal FHR (bpm) *mean (SD)*	17	140 (10)	140 (10)	1.000
Accelerations (*n*) *median (IQR)*	17	9 (3 to 15)	9 (3 to 15)	0.157
Sporadic decelerations (*n*) *median (IQR)*	17	3 (2 to 11)	3 (1 to 8)	0.317
Prolonged decelerations (*n*) *median (IQR)*	17	0 (0 to 0)	0 (0 to 0)	1.000
Abnormal LTV (%) *median (IQR)*	17	3 (0 to 7)	3 (1 to 11)	0.317
Alarm *n (%)*	17			1.000
Green-Blue		14 (82)	14 (82)	
Yellow-red		3 (18)	3 (18)	
Major MHR-FHR ambiguity				
Signal loss (%) *mean (SD)*	44	22 (11)	28 (13)	**<0.001**
Basal FHR (bpm) *mean (SD)*	44	136 (13)	136 (12)	0.129
Accelerations (*n*) *median (IQR)*	44	7 (4 to 10)	6 (3 to 10)	0.285
Sporadic decelerations (*n*) *median (IQR)*	44	8 (6 to 10)	6 (4 to 10)	**0.001**
Prolonged decelerations (*n*) *median (IQR)*	44	0 (0 to 1)	0 (0 to 0)	**0.046**
Abnormal LTV (%) *median (IQR)*	44	1 (0 to 3)	1 (0 to 3)	0.414
Alarm *n (%)*	44			**0.021**
Green-Blue		15 (34)	23 (52)	
Yellow-red		29 (66)	21 (48)	
Overall				
Signal loss (%) *mean (SD)*	61	20 (11)	24 (13)	**<0.001**
Basal FHR (bpm) *mean (SD)*	61	137 (12)	137 (12)	0.129
Accelerations (*n*) *median (IQR)*	61	7 (4 to 10)	7 (3 to 10)	0.170
Sporadic decelerations (*n*) *median (IQR)*	61	7 (4 to 10)	6 (3 to 9)	**0.001**
Prolonged decelerations (*n*) *median (IQR)*	61	0 (0 to 1)	0 (0 to 0)	**0.046**
Abnormal LTV (%) *median (IQR)*	61	1 (0 to 4)	2 (0 to 5)	0.197
Alarm *n (%)*				**0.021**
Green-Blue		29 (48)	37 (61)	
Yellow-red		32 (52)	24 (39)	

*SD* standard deviation, *IQR* inter-quartile range

*p* values with statistical significance are displayed in bold

**Table 3:** Changes in overall FHR classification before and after the removal of MHR-FHR ambiguities, in acidemic and non-acidemic cases (umbilical artery blood pH <7.15 or ≥7.15)

	Before *n* (%)	After *n* (%)	*P*
Acidemic newborns (*n* = 7)			1.000
Green-Blue	3 (57)	3 (57)	
Yellow-red	4 (43)	4 (43)	
Non-acidemic newborns (*n* = 54)			**0.021**
Green-Blue	26 (48)	34 (63)	
Yellow-red	28 (52)	20 (37)	

*p* values with statistical significance are displayed in bold

For statistical analysis, the results were expressed as medians (with inter-quartile ranges), for skewed continuous variables. The Mann–Whitney test was used to compare the groups with minor or major ambiguities (Table 1) and the Wilcoxon sign rank was used to compare the classification results before and after removal of ambiguities (Table 2). For other continuous variables, results were expressed as means and standard deviations. The independent sample *t* test was used to compare the groups with minor or major ambiguities (Table 1) and the paired *t* test was used to evaluate results before and after removal of ambiguities (Table 2). To compare categorical variables between minor or major ambiguity groups (Table 1) and before and after ambiguity removal (Tables 2 and 3), the Chi-square (or Fisher exact test) and McNemar tests were used, respectively. Ninety-five percent confidence intervals were calculated as appropriate and p-values considered significant at less than 0.05.

## Results

The general maternal, fetal, delivery and newborn characteristics, in relation with the presence of minor and major MHR-FHR ambiguities, are presented in Table 1. Overall, average maternal body mass index and age were, respectively, 27 Kg/m^2^ (SD:3) and 28 years (SD:5). Average gestational age was 39 weeks (SD:1). Twenty-six percent of women were multiparous and all fetuses were in cephalic presentation. The average systolic and diastolic blood pressures were, respectively, 122 (SD:11) and 74 mmHg (SD:8) and the average axillary temperature was 36.2 °C (SD:0).

C-section was performed in 10 % of the group and instrumental vaginal delivery in 38 %. All C-sections were performed during the first stage of labor because of protracted or arrested labour. Forty-four percent of newborns were males, with an average one and five-minute Apgar score of 9 (range: 9–10) and 10 (range:10–10), respectively. The average umbilical artery blood pH was 7.24 (SD:0.07). The average length of the active phase of labour was 297 min (SD:170).

Five tracings did not exhibit any MHR-FHR ambiguity, 12 exhibited 1 % of ambiguities and 44 (72 %) exhibited major MHR-FHR ambiguities (11 tracings with 2 %, 7 with 3 %, 5 with 4 % and 21 with 6–33 %).

In the group with minor MHR-FHR ambiguities, maternal, fetal, delivery and newborn characteristics were similar in the five cases that did not exhibit any ambiguity compared with the 12 cases that exhibited 1 % of ambiguities. Major ambiguities were significantly associated with MHR accelerations, FHR signal loss, decelerations and yellow or red alarms, when compared with minor ambiguities (Table 1).

Removal of ambiguities resulted in a significant increases in FHR signal loss, both in cases with minor and major ambiguities. When major ambiguities occurred, a decrease in the number of FHR decelerations, and a decrease in the false positive rate of yellow-red alarms for the detection of acidemic fetuses, were also observed, but this was not seen with minor ambiguities (Tables 2 and 3).

## Discussion

In this study, 72 % of tracings acquired in the last recorded hour of labour exhibited episodes of major MHR-FHR ambiguity. This concurs with the report by Reinhard et al. [1] showing that MHR-FHR ambiguities with clinical significance may be much more frequent and subtle than suggested by anecdotal reports published in the literature, most of them confined to extreme cases of fetal acidemia or death [2–8].

Another finding in the present study is that MHR-FHR ambiguities should not only be suspected when accelerations coincide with uterine contractions [2–8, 10], but also when FHR decelerations occur in association with signal loss (Table 1 and Fig. 1). Most MHR tracings are characterized by repetitive accelerations, which may overlap FHR signals and mimic FHR decelerations or baseline segments during episodes of FHR signal loss [15, 20, 21] (Fig. 1). With the high epidural rate observed in this population, it is possible that there was a lower increase in MHR during active pushing, and therefore only a few cases of suspected FHR accelerations due to inadvertent MHR recording were detected. Only 7 % cases had a MHR baseline above 100 bpm, and in no cases it exceeded 110 bpm. MHR accelerations rarely reached the FHR baseline and this resulted in frequent MHR-FHR ambiguities being caused by MHR baseline segments being confused with FHR decelerations (Fig. 1). It may be almost impossible to detect subtle misinterpretations without computer analysis of simultaneous MHR and FHR recordings [20], with automated detection of ambiguities.

C-sections were not evaluated separately as risk factor of MHR-FHR ambiguities because of the small number of cases. However, it was observed that C-sections were associated with fewer MHR-FHR ambiguities, although without assessing statistical significance. This probably occurred because C-sections were performed predominantly during the first stage of labour. The second stage is characterized by higher signal loss, more frequent MHR accelerations and FHR decelerations. Further studies to assess if C-section is an independent risk factor for major MHR-FHR ambiguities are warranted.

To our knowledge, the effect of systematic removal of MHR-FHR ambiguities has not been previously assessed. In this study, removal of ambiguities resulted in a significant increase in FHR signal loss, along with a decrease in the number of FHR decelerations and an improvement in tracing classification (Tables 2 and 3). Again, the improvements were more often related with the misinterpretation of FHR decelerations than accelerations. If our results are confirmed in larger studies, it may be possible to decrease the unnecessary intervention associated with false positive yellow-red alarm in non-acidemic fetuses, without compromising sensitivity.

Several limitations need to be considered in this study and should be taken in consideration of future research. Firstly, a higher-risk population needs to be evaluated, as the number of cases with umbilical artery pH under 7.15 was low, and this 10th percentile cut-off value has limited clinical significance [22, 23]. Secondly, other methods for simultaneous MHR signal acquisition need to be evaluated, namely those using oximetry. Thirdly, the criterion used for removal of MHR-FHR ambiguities [1] may benefit from refinement after analysis of cases with adverse perinatal outcome, ideally using simultaneous internal FHR monitoring as the gold standard for fetal signals.

In conclusion, removal of MHR-FHR ambiguities resulted in a significant decrease in FHR decelerations and an improvement in tracing classification. Larger studies are needed to confirm the impact of this methodology on the diagnostic accuracy of intrapartum cardiotocography.
